# Heptamethine Cyanine Dye-Doped Single-Walled Carbon Nanotube Electrodes for Improving Performance of HTL-Free Perovskite Solar Cells

**DOI:** 10.3390/molecules30010060

**Published:** 2024-12-27

**Authors:** Man-Ge Cai, Arina Watanabe, Zhenyu Xu, Yong-Chang Zhai, Achmad Syarif Hidayat, Naoki Ueoka, Miftakhul Huda, Kimitaka Higuchi, Esko I. Kauppinen, Kazumasa Funabiki, Yutaka Matsuo

**Affiliations:** 1 Department of Chemical Systems Engineering, Graduate School of Engineering, Nagoya University, Nagoya 464-8603, Japanmiftakhul.huda.j7@f.mail.nagoya-u.ac.jp (M.H.); 2 Department of Chemistry and Biomolecular Science, Gifu University, Gifu 501-1193, Japan; 3 Department of Applied Physics, School of Science, Aalto University, 00076 Espoo, Finland; zhenyu.xu@aalto.fi; 4 High Voltage Electron Microscope Laboratory, Institute of Materials and Systems for Sustainability, Nagoya University, Nagoya 464-8603, Japan; 5 Institute of Materials Innovation, Institutes for Future Society, Nagoya University, Nagoya, 464-8603, Japan

**Keywords:** single-walled carbon nanotubes, hole-transporting layer free, heptamethine cyanine dye, perovskite solar cells, metal-free, carbon electrode

## Abstract

Perovskite solar cell (PSC) technology holds great promise with continuously improving power conversion efficiency; however, the use of metal electrodes hinders its commercialization and the development of tandem designs. Although single-walled carbon nanotubes (SWCNTs), as one-dimensional materials, have the potential to replace metal electrodes in PSCs, their poor conductivity still limits their application. In this study, the near-infrared (NIR)-absorbing anionic heptamethine cyanine dye-doped SWCNTs functioned in a dual role as an efficient charge-selective layer and electrode in PSCs. Benefiting from the improvement in conductivities and matched energy level of doped-SWCNT, the dual-role SWCNT electrodes applied to PSCs achieved a better performance than the undoped PSCs with a higher short circuit current (*J*_SC_) and fill factor (FF).

## 1. Introduction

Perovskite solar cells (PSCs) have demonstrated the potential to play an important role in promising photovoltaic cells for commercialization, providing the benefits of a low manufacturing cost and high photoelectric conversion efficiency [1,2,3,4,5]. Although a power conversion efficiency (PCE) of over 25% can be achieved owing to the structural engineering and the passivation treatment [6,7,8], the limitations in long-term stability along with the utilization of expensive hole transport layer (HTL) materials and noble metal electrodes are obstacles to the commercialization of PSCs [9,10,11].

Single-walled carbon nanotubes (SWCNTs) are recognized as a promising material for replacing the metal electrodes in PSCs due to their massive production volumes [12,13], their mechanical flexibility [14,15] and their prevention of ion migration [16]. Tremendous efforts have been devoted to applying the CNTs in PSCs as electrodes to facilitate commercialization [17,18,19,20]. Favorably, when used as p-type semiconductors, SWCNTs can block the electronics and collect the holes. When the SWCNTs were treated with p-dopants, they usually gave a better performance [18,19,20]. However, some p-dopants used for SWCNTs are troubling with a high volatility (e.g., HNO_3_) that results in a degradation in performance over time. Some expensive hole transport layer (HTL) materials such as Spiro-OMeTAD or poly(triarylamine) (PTAA) also work as dopants. Although certain results can be achieved with these materials, their use is detrimental to promoting the commercialization of PSCs owing to their high synthetic cost, thus the HTL-free PSCs with carbon electrodes are necessary. Cyanine dyes (Cy dyes), as successful industrialization compounds, have gained considerable attention in biology [21,22,23], particularly in dye-sensitized solar cells [24,25,26,27] and photodetectors [28,29,30]. The near-infrared absorption properties of cyanine dyes do not affect perovskite light absorption, which makes them good dopant candidates for SWCNTs.

Herein, we report that, as a p-dopant, the near-infrared (NIR)-absorbing anionic heptamethine cyanine (HMC) dye, namely GNW-5, improves the conductivity and the Fermi energy of SWCNTs without losing the light absorption of perovskite. Their better hole transporting mobility and better energy level alignments with perovskite make GNW-5-doped, SWCNT-based PSCs have a higher short circuit current density (*J*_SC_) and fill factor (FF), thus resulting in better performance.

## 2. Results and Discussion

**Figure 1:**
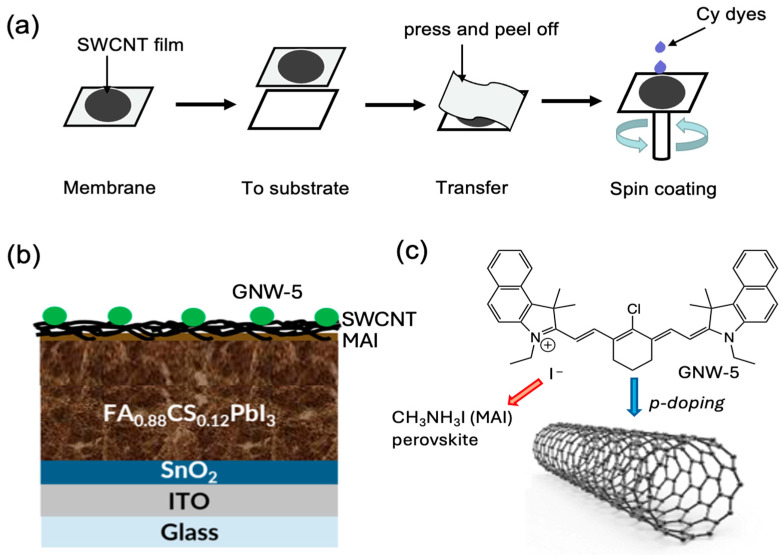
Concept of GNW-5-doped SWCNTs utilized as electrodes in PSCs. (**a**) Doping GNW-5 dye molecules using spin-coating. (**b**) Perovskite solar cell structure used for this work. (**c**) Schematic diagram of the molecular structure of GNW-5 and its interaction with SWCNTs.

The SWCNTs and GNW-5 employed in this paper were synthesized based on the previous literature [31,32]. The diameters (*d*) of synthesized SWCNTs were determined to be 1.8 nm by measuring the radial breathing mode (ω_RBM_) of the Raman spectra according to the formula *d* = 248/ω_RBM_ (Figure S1). To assess the performance of GNW-5-doped SWCNTs as electrodes in PSCs, perovskite solar cells with an inverted structure ITO/SnO_2_ (tin dioxide)/FA_0.88_Cs_0.12_PbI_3_/MAI (Methylammonium iodide)/SWCNT were fabricated (Figure 1a,b). The SWCNT film deposited on the membrane was attached to the MAI layer by pressing and peeling off the membrane. Thanks to the fiber-like structure of the SWCNTs, the GNW-5 can be easily doped on the electrode by spin-coating (Figure 1a).

**Figure 2:**
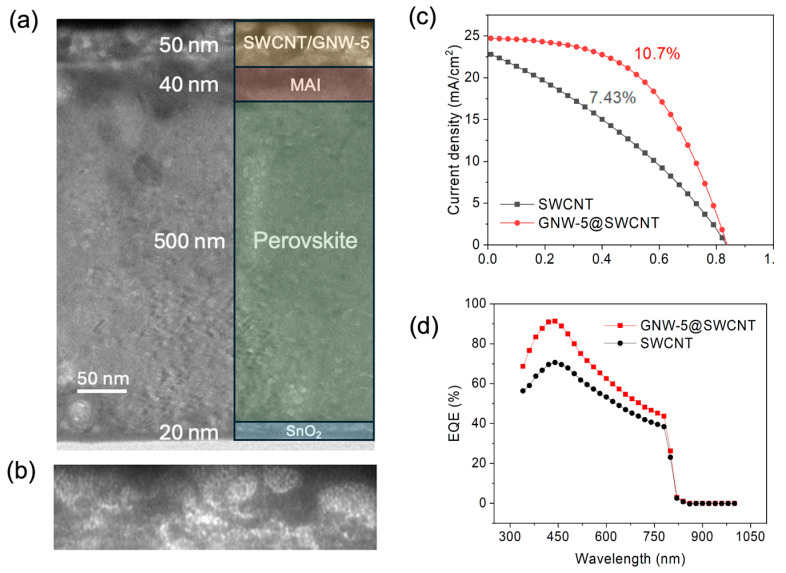
Cross-sectional TEM images of (**a**) GNW-5-doped PSCs and (**b**) detailed enlargement of the GNW-5-doped SWCNT layer. (**c**) *J–V* curves of PSCs using GNW-5-doped SWCNTs and pristine SWCNTs. (**d**) EQE spectra of PSCs before and after doping with GNW-5.

Cross-section transmission electron microscopy (TEM) was applied to determine the layer thickness of the fabricated device (Figure 2a,b). A 500 nm perovskite layer can be observed, which ensures sufficient light absorption. The GNW-5-doped SWCNT was observed to be 50 nm without changing the fiber-like structure of SWCNT substantially (Figure 2b). Then, the current density–voltage (*J*–*V*) curves of the SWCNT electrodes applied to the PSCs using GNW-5 were evaluated. The pristine SWCNT device showed a PCE of 7.43% with a *J*_SC_ of 22.68 mA/cm^2^, a *V*_OC_ of 0.84 V, and an FF of 0.38. The introduction of GNW-5 to the SWCNT improved the solar cell output performance significantly with a higher PCE of 10.52%, a *J*_SC_ of 24.7 mA/cm^2^, a *V*_OC_ of 0.84 V, and an FF of 0.52 (Figure 2c). It is worth noting that the GNW-5-doped device shows a lower series resistance (R_s_) and a higher shunt resistance (R_sh_, Figure S2). The smaller R_s_ reveals better contact between the doped SWCNT and the electrode, ensuring a better FF (Figure S3). The external quantum efficiency (EQE) spectra indicated that the GNW-5-doped SWCNT electrodes in PSCs have a higher conversion efficiency across a range from 300 nm to 800 nm than pristine SWCNT electrodes in PSCs, which is in accordance with the higher *J*_SC_ value of the GNW-5-doped SWCNT electrodes in PSCs (Figure 2d). To evaluate the effect of the concentration of GNW-5 on the performance of the PSCs, the SWCNT electrode was treated with GNW-5 under different concentrations. The concentration of dopant mainly affected the *J*_SC_ and FF (Table 1). We found that either too high or too low of a dopant concentration will lead to a decrease in the FF and *J*_SC_, but it has almost no effect on the *V*_OC_.

To better understand the reason for the performance improvement seen with the GNW-5-doped SWCNT electrodes in PSCs, characterizations of the GNW-5-doped SWCNTs were performed. Transmittance is a crucial factor affecting the performance of PSCs that is explained by the fact that high transmittance ensures sufficient light can be collected and converted by the perovskites. The GNW-5-doped SWCNTs exhibited a lower sheet resistance and a higher transmittance (Table 2). The introduction of anionic heptamethine cyanine greatly improved the conductivity of the SWCNT electrode, thus reducing the series resistance of the device and affording a higher *J*_SC_ value.

**Figure 3:**
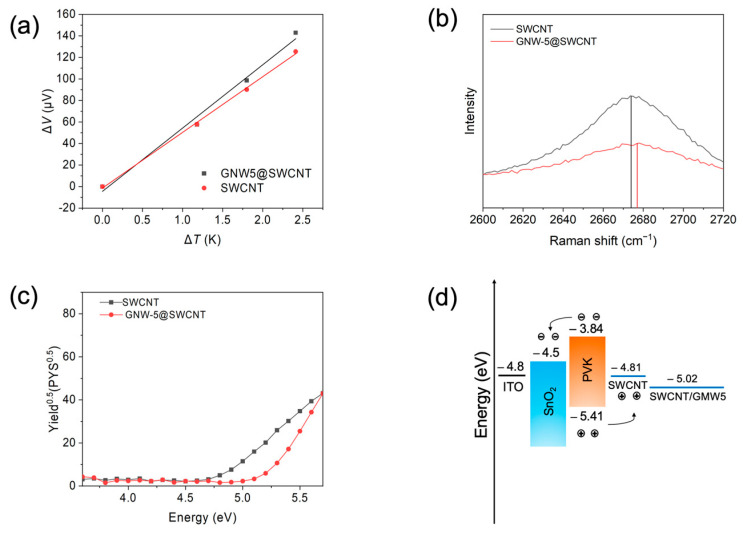
Properties of SWCNT before and after GNW-5 doping. (**a**) Seebeck coefficient measurement with fitting curve. (**b**) 2D peak maximum of Raman spectrum. (**c**) Photoelectron yield spectroscopy. (**d**) Energy level diagram of PSCs with GNW-5 doping.

The Seebeck coefficient (*S*) measurement was conducted to gain further insights into the doping effectiveness of GNW-5. By measuring the temperature difference and the voltage difference between two points on the SWCNT, one can easily know the majority of the carrier characteristics of the SWCNT based on the equation *S* = ∆*V*/∆*T*, as the Seebeck coefficient is usually dominated by the contributions of charge carrier diffusion, which tends to push the charge carriers to the colder side of the SWCNT until a compensation voltage is established. GNW-5-doped SWCNTs exhibited stronger p-type semiconductor characteristics than pristine SWCNTs, with a Seebeck coefficient of 87.96 μV (Figure 3a). The doping effect can also be observed on the Raman spectrum. Increasing the D-band to G-band of intensity ratio (*I*_D_/*I*_G_) from 0.063 to 0.133 indicates the success of the doping (Figure S4). The position of the 2D peak maximum on the Raman spectrum also provides important information about doping (Figure 2b). A blue shift of the 2D peak maximum of the SWCNT was observed after doping, indicating the p-doping effect of GNW-5. The UV–vis absorption spectrum was then obtained. There is a 35 nm red shift of π-π* transition seen with the GNW-5-doped SWCNT, from 278 nm to 313 nm, which is due to the p-π conjugation formed between the GNW-5 and SWCNTs, and improves the conductivity of the SWCNT electrode (Figure S5) [33]. Photoelectron yield spectroscopy (PYS) was then carried out to investigate the energy levels of the GNW-5-applied SWCNT electrode film (Figure 3c). The pristine SWCNT film showed the highest occupied molecular orbital (HOMO) energy level of −4.81 eV. When the SWCNT was doped with the GNW-5, the HOMO energy level was up-shifted to −5.02 eV, which has a better band alignment with the valence band of perovskite, ensuring a larger FF (Figure 3d). We anticipate that the counter anion, iodide, may have had a desirable effect on the perovskite layer, similar to the added MAI.

Finally, the steady-state photoluminescence (PL) quenching spectra was then measured with the structure ITO/PVK/SWCNT (GNW-5) to assess the hole extraction ability of the SWCNT (Figure S6). The GNW-5-doped SWCNT gives more effective hole transporting mobility than the pristine SWCNT, which provides the benefit of a higher *J*_SC_ and FF. The improved performance of the GNW-5-applied SWCNT electrode-based PSCs might also be due to the fact that the GNW-5 can also work as a hole transport layer (HTL), which contributes to the effectiveness of the electronic blocking and hole transportation. Finally, the durability of the GNW-5 doping effect was determined by examining the changes in sheet resistance of SWCNT film. Some p-dopants used for SWCNTs are troubling with the high volatility, the doping effect are decreasing as it evaporates. GNW-5-doped SWCNTs can maintain the conductivity for 720 h without any reduction (Figure S7). The higher performance and the long durability of GNW-5-doped SWCNT are conducive to promoting the commercialization of PSCs in the future.

## 3. Materials and Methods

### 3.1. Materials

Unless otherwise noted, all materials including dry solvents were obtained from commercial suppliers (Sigma-Aldrich, St. Louis, MO, USA, TCI Tokyo Japan, Wako, Tokyo, Japan) and used without further purification.

### 3.2. SWCNT Preparation

The synthesis condition employed in this research was adapted from a previously reported method [34]. Ferrocene and thiophene (2 wt.%, Fe/S molar ratio of 2.5) were dissolved in 10 mL of toluene, sonicated for 1 min, and loaded into a 10 mL glass syringe with a Teflon piston tip. Using a syringe pump (NE-1000 series, New York, NY, USA), the solution was injected at 0.6 mL/h into a floating catalyst chemical vapor deposition (FC-CVD) system.

The solution was evaporated at 130 °C and the precursor vapor was carried by a gas flow of 1.5 slm (standard liters per minute) H_2_ and 10 slm N_2_. The furnace temperature was maintained at a temperature of 1180 °C. Single-walled carbon nanotubes (SWCNTs) were collected onto a membrane filter at the furnace outlet. The resulting SWCNT film can be transferred to a target substrate for further characterization.

### 3.3. FA_0.88_Cs_0.12_PbI_3_ Solution Preparation

A total of 116.3 mg CH_4_N_2_·HI, 23.4 mg CsI, 380.3 mg PbI_2_ and 75 μL anhydrous dimethyl sulfoxide were mixed with 425 μL anhydrous *N*,*N*-dimethylformamide and stored in a nitrogen-filled glove box at room temperature for 12 h to fully dissolve. The solution was filtered through a 0.20 μm poly(tetrafluoroethylene) filter before use.

### 3.4. SnO_2_ Solution Preparation

A total of 100 μL tin (IV) oxide (15% solution in H_2_O colloidal dispersion) was diluted with 500 μL deionized water. The mixed solution was sonicated for 15 min and filtered through a 0.20 μm poly(tetrafluoroethylene) filter before use.

### 3.5. Device Fabrication

ITO-coated glass (sheet resistance ≤ 10 Ω cm^2^) was cleaned consecutively in detergent, deionized water, acetone, isopropanol, and ethanol ultrasonic baths for 15 min, respectively. Then, the cleaned ITO-substrates underwent UV–ozone for 15 min for enhancement of wettability and removal of any organic contamination. The filtered SnO_2_ solution was used to deposit a dense SnO_2_ layer. A total of 40 μL SnO_2_ solution was spin-coated on the clean ITO substrate at a rotation speed of 3000 rpm for 30 s. The spin-coated film was annealed with hot plate at 150 °C for 30 min before transferring to the glove box. Before spin-coating the prepared perovskite solution, the SnO_2_-coated film was treated with a UV–ozone device for 15 min to enhance wettability in preparation for the subsequent spin-coating of the perovskite layer. Then, 30 μL of perovskite precursor solution was deposited on the SnO_2_ layer by spin-coating at a speed of 4000 rpm for 30 s. A total of 8 s after the spin-coating process started, 150 μL of chlorobenzene was dropped onto the substrate. After the spin-coating was completed, the film was transferred to the hot plate and annealed at 110 °C for 20 min. After annealing, 30 μL of MAI solution was coated on the perovskite layer at a speed of 4000 rpm for 30 s, then annealed on a hot plate at 100 °C for 1 min. On the prepared ITO/SnO_2_/FA_0.88_CS_0.12_PbI_3_/MAI/substrate, the pressure transfer method was applied on the cooled perovskite surface to reduce the T-70% transparency re-cut carbon nanotube film was laminated on top. A few drops of chlorobenzene were placed on top of the carbon nanotube film to increase contact between the carbon nanotubes and the perovskite layer. Finally, the prepared device was transferred to a hot plate to treat at 80 °C for 1 min to evaporate the dropped chlorobenzene.

### 3.6. Device Characterization

The *J*−*V* curves were measured using a software-controlled source meter (HAL-C-100, Asahi Spectroscopy Co., Ltd., Tokyo, Japan) and simulated sunlight was shined onto a solar irradiance meter connected to a galvanometer. In order to adjust the radiation intensity E to E = 100 mW/cm^2^, the height of the simulated sunlight was adjusted so that the voltage was 55.4 V, and the *J*−*V* characteristics of the device were measured at this height. A 0.04 cm^2^ mask was placed on the glass substrate and light entered from ITO side. A four-probe sheet resistance tester (SR-H1000C. HiSOL, Inc., Tokyo, Japan) was used to test the changes in sheet resistance of SWCNT films to analyze changes in conductive properties. The Focused Ion Beam System (Hitachi High-Tech Corp. FB-2100. Ibaraki, Japan) was used to accurately cut the sample and prepare a sample specifically for TEM. The TEM analysis of device cross-section was performed using Hitachi High-Tech Corp. FB-2100. A ZEM-2 (ADVANCE RIKO, Inc. ZEM-2. Ibaraki, Japan) was used to detect the change in the Seebeck coefficient of the SWCNT films to determine the doping effect of the dye molecules. An inVia Raman microscope (Renishaw, West Dundee, IL, USA) was employed for the vibrational spectra observation of SWCNTs and HTL-applied SWCNTs with 532 nm laser wavelength (JASCO Corporation, Tokyo, Japan). The PL measurements were performed using JASCO Spectrofluorometer (FP-6600. Tokyo, Japan). The work function measurements of SWCNTs were performed using Photoelectron Yield Spectroscopy in Air (Riken Keiki AC-2. Tokyo, Japan). The optical absorption spectra of pristine SWCNT film was measured using UV-Vis-NIR spectrometer (JASCO·MSV-5200. Tokyo, Japan).

## 4. Conclusions

In conclusion, we achieved high efficiencies in the perovskite solar cells with transparent single-walled carbon nanotube-based electrodes, utilizing GNW-5 as a dopant. The introduction of GNW-5 greatly improved the conductivity of the SWCNT electrodes. The doped SWCNTs also show an energy level structure that is in better alignment with the HOMO energy level of the perovskite. The mechanism of improving the *J*_SC_ and FF was then investigated, providing valuable insights into using dopants for SWCNT electrode treatments. Therefore, this study not only established the methodology for applying dye dopants on SWCNT-based electrodes in PSCs, but it also paves the way for the development and commercialization of PSCs using SWCNT-based electrodes.

## Figures and Tables

**Table 1:** The perovskite solar cell performance of different concentration of GNW-5.

Dopants	*J*_SC_ [mA/cm^2^]	*V*_OC_ [V]	FF [-]	PCE [%]
GWN-5 0.25 mg/mL	23.60	0.82	0.51	9.82
GWN-5 0.50 mg/mL	24.71	0.84	0.52	10.70
GWN-5 1.00 mg/mL	23.71	0.82	0.49	9.55
Pristine SWCNT	22.68	0.84	0.38	7.43

**Table 2:** Transmittance and sheet resistance changes before and after GNW-5 doping.

CNTs	Sheet Resistance [Ω/sq]	Transmittance [%]
GNW-5-doped SWCNT	107	70 ^a^
Pristine SWCNT	156	65 ^a^

^a^ Transmittance was measured at 550 nm.

## Data Availability

All relevant data are contained within the manuscript. The data that support the findings of this study are available in the ESI^†^ of this manuscript.

## Supplementary Materials

The following supporting information can be downloaded at: https://www.mdpi.com/article/10.3390/molecules30010060/s1, Figure S1: Raman spectrum of the radial breathing mode region of SWCNTs film used in this paper; Figure S2: J–V curves of PSCs using GNW-5 doped SWCNT and pristine SWCNT; Figure S3: The performance distributions of PSCs with different electrode; Figure S4: Raman spectrum of SWCNTs before and after GNW-5 doping; Figure S5: Uv-vis absorption spectrum of SWCNT before and after GNW-5 doping; Figure S6: The steady-state photoluminescence (PL) quenching spectra of SWCNT before and after doping with the structure ITO/PVK/SWCNT (GNW-5); Figure S7: The sheet resistance changes over time of SWCNT before and after doping.
