# Supplementary material for: Heptamethine Cyanine Dye-Doped Single-Walled Carbon Nanotube Electrodes for Improving Performance of HTL-Free Perovskite Solar Cells
Source: Molecules. 2024 Dec 27;30(1):60. doi: 10.3390/molecules30010060 (PMC11721129; doi:10.3390/molecules30010060)
Supplement: Supplementary file 1 [file molecules-30-00060-s001.zip › molecules-3382540-supplementary.pdf]

Supporting Information

Heptamethine cyanine dye-doped single-walled carbon nano-tubes electrode for improving the performance of HTL-free perovskite solar cells

Man-Ge Cai [1], Arina Watanabe [2], Zhenyu Xu [3], Yong-Chang Zhai [1], Achmad Syarif Hidayat [1], Naoki Ueoka [1], Miftakhul Huda [1], Kimitaka Higuchi [4], Esko I. Kauppinen [3,*], Kazumasa Funabiki [2,*], and Yutaka Matsuo [1,5,*]

1. Department of Chemical Systems Engineering, Graduate School of Engineering, Nagoya University, Nagoya 464-8603, Japan
2. Department of Chemistry and Biomolecular Science, Gifu University, Gifu, 501-1193 Japan
3. Department of Applied Physics, Aalto University School of Science, PO Box 15100, FI-00076, Finland
4. High Voltage Electron Microscope Laboratory, Institute of Materials and Systems for Sustainability, Nagoya University, Nagoya 464-8603, Japan
5. Institute of Materials Innovation, Institutes for Future Society, Nagoya University, Nagoya, 464-8603, Japan

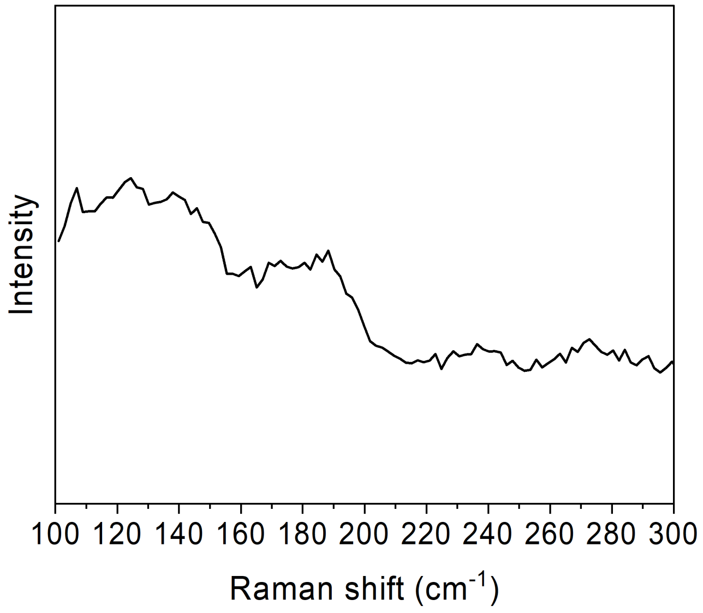

Figure S1. Raman spectrum of the radial breathing mode region of SWCNTs film used in this paper.

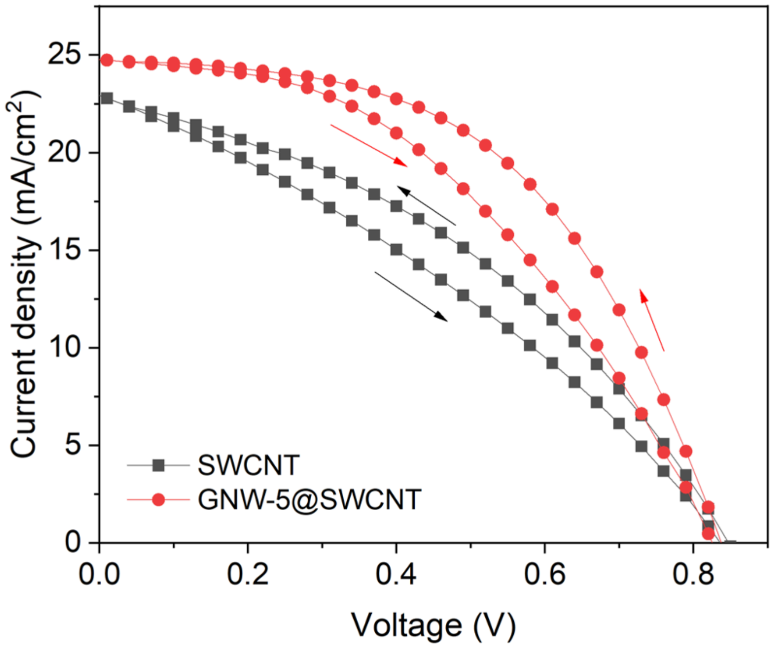

Figure S2. *J*–*V* curves of PSCs using GNW-5 doped SWCNT and pristine SWCNT.

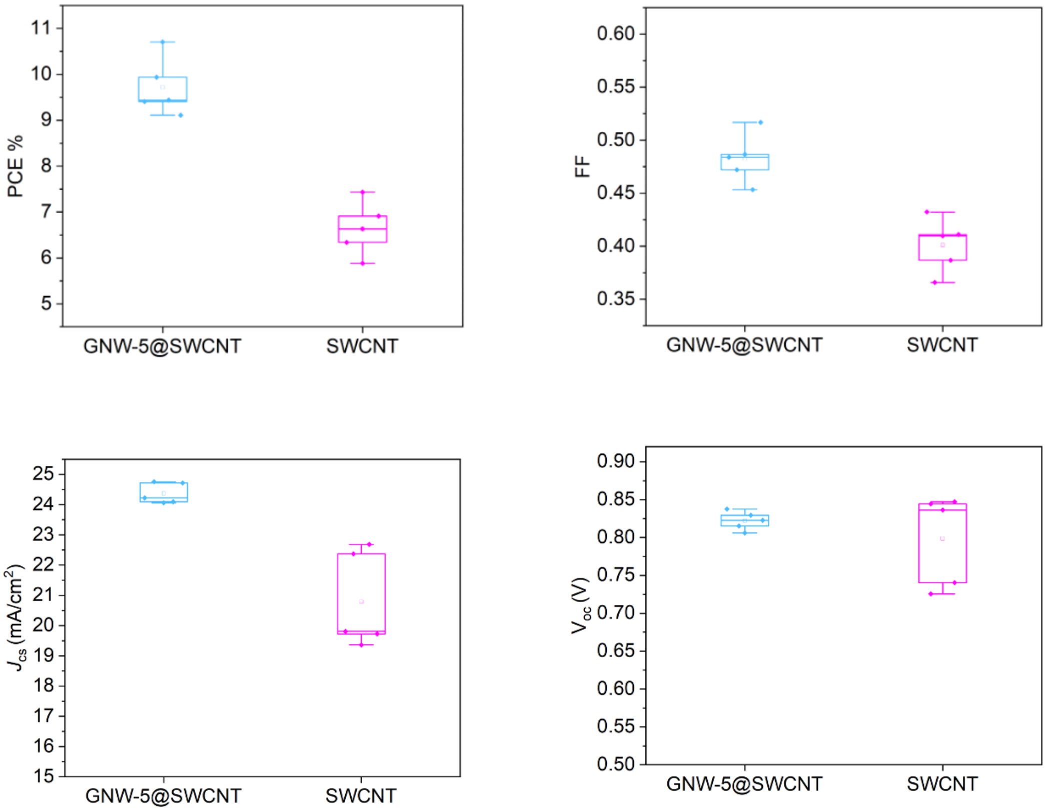

Figure S3. The performance distributions of PSCs with different electrode.

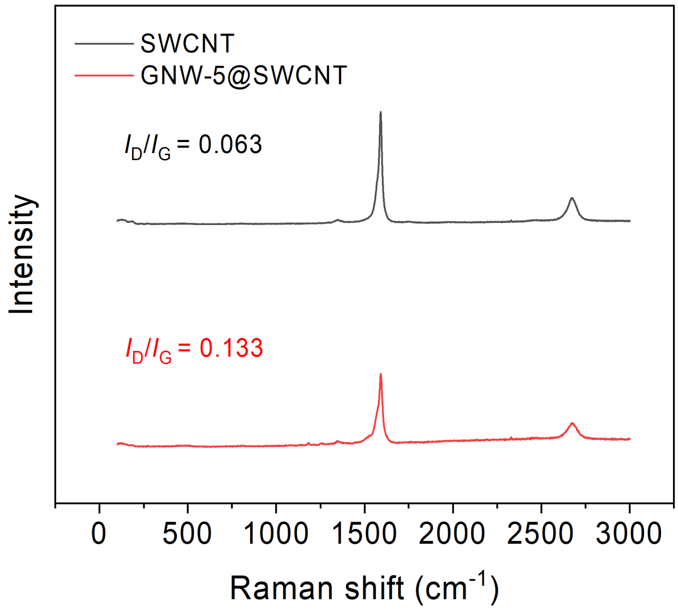

Figure S4. Raman spectrum of of SWCNTs before and after GNW-5 doping.

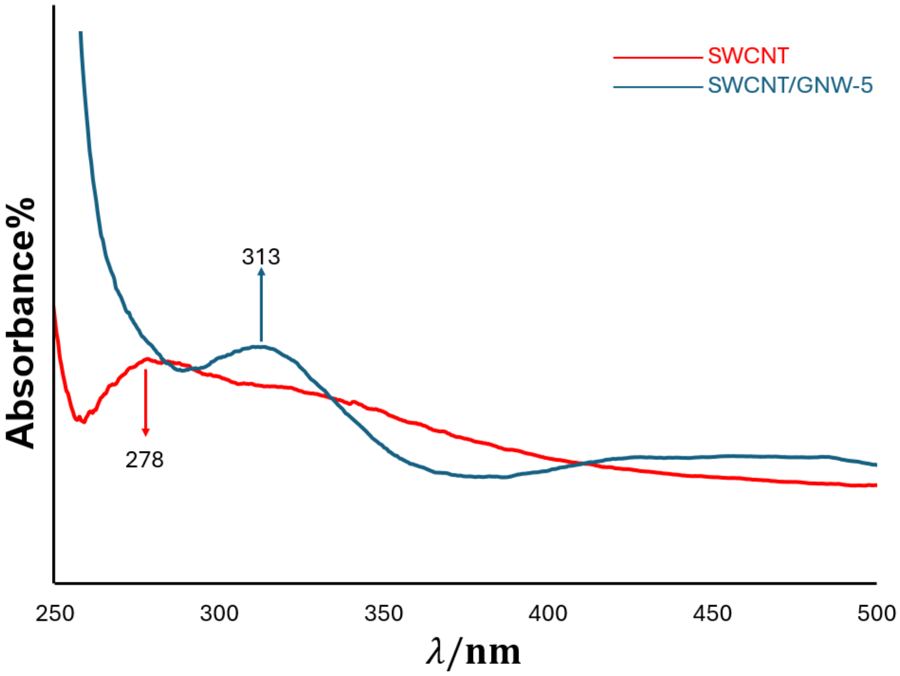

Figure S5. Uv-vis absorption spectrum of SWCNT before and after GNW-5 doping.

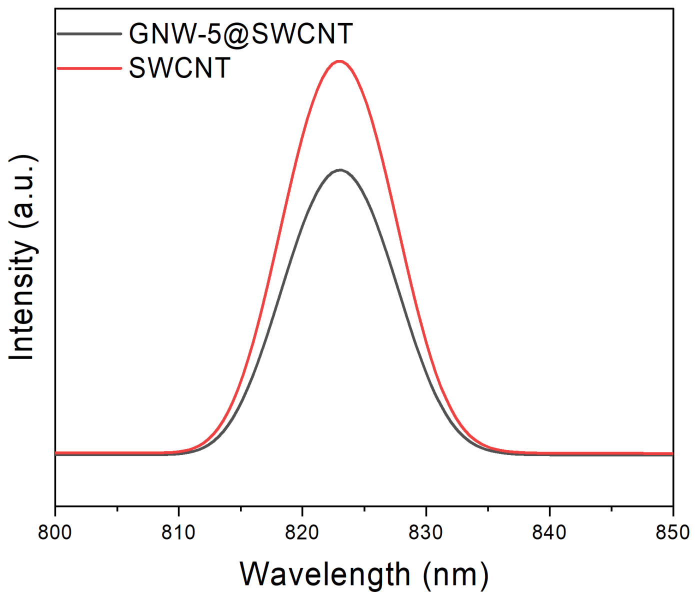

Figure S6. The steady-state photoluminescence (PL) quenching spectra of SWCNT before and after doping with the structure ITO/PVK/SWCNT (GNW-5).

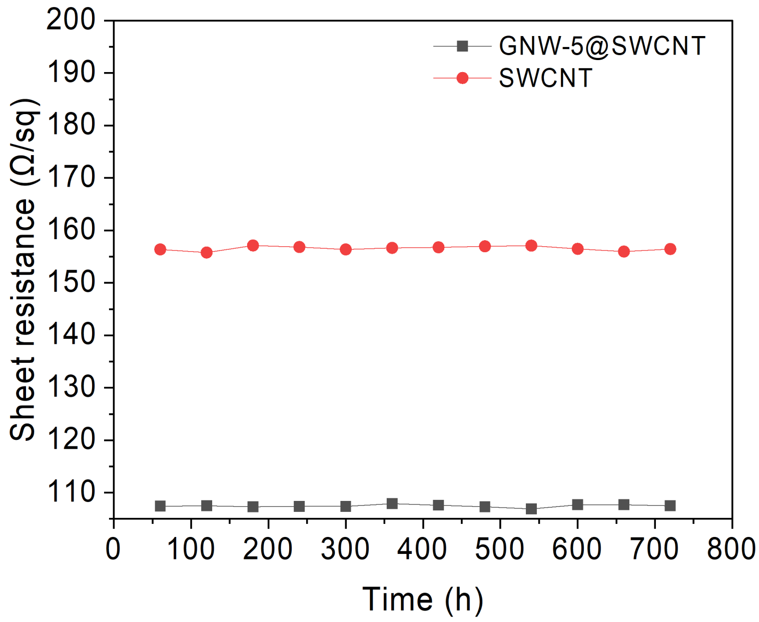

Figure S7. The sheet resistance changes over time of SWCNT before and after doping.
